# Multi-Layer Polyurethane-Fiber-Prepared Entangled Strain Sensor with Tunable Sensitivity and Working Range for Human Motion Detection

**DOI:** 10.3390/polym16081023

**Published:** 2024-04-09

**Authors:** Weibing Zhong, Daiqing Wang, Yiming Ke, Xiaojuan Ming, Haiqing Jiang, Jiale Li, Mufang Li, Qianqian Chen, Dong Wang

**Affiliations:** 1 Key Laboratory of Textile Fiber and Products, Ministry of Education, Wuhan Textile University, Wuhan 430200, China; weibingzhong09@gmail.com (W.Z.); dave827513984@163.com (D.W.); 2015040@wtu.edu.cn (H.J.); 13667212217@163.com (J.L.); limufang223@126.com (M.L.); 2 College of Chemistry and Chemical Engineering, Donghua University, Shanghai 201620, China; keymingwtu@163.com (Y.K.); xiaojuanming@163.com (X.M.); 3 Department of Physical Education, Wuhan Textile University, Wuhan 430200, China

**Keywords:** polyurethane, entangled fiber sensor, strain sensor, human motion detection

## Abstract

The entanglement of fibers can form physical and topological structures, with the resulting bending and stretching strains causing localized changes in pressure. In this study, a multi-layer polyurethane-fiber-prepared (MPF) sensor was developed by coating the CNT/PU sensing layer on the outside of an elastic electrode through a wet-film method. The entangled topology of two MPFs was utilized to convert the stretching strain into localized pressure at the contact area, enabling the perception of stretching strain. The influence of coating mechanical properties and surface structure on strain sensing performance was investigated. A force regulator was introduced to regulate the mechanical properties of the entangled topology of MPF. By modifying the thickness and length proportion of the force regulator, the sensitivity factor and sensitivity range of the sensor could be controlled, achieving a high sensitivity factor of up to 127.74 and a sensitivity range of up to 58%. Eight sensors were integrated into a sensor array and integrated into a dance costume, successfully monitoring the multi-axis motion of the dancer’s lumbar spine. This provides a new approach for wearable biomechanical sensors.

## 1. Introduction

Wearable sensors have distinct advantages in the monitoring of physiological signals and daily activities and have been successfully applied in medical rehabilitation and patient care. The inertial sensors, bioelectrical sensors, and biomechanical sensors, combined with specific algorithms, are mainly employed to acquire and reconstruct human activity information [1,2,3,4,5,6]. However, the rigid inertial sensors compromise comfort for long-term wearability, with bioelectrical sensors posing high demands on the circuit’s amplification capability and the adaptability of the algorithms. The biomechanical sensors, by incorporating sensing functionality into textiles, achieve a balance between sensing and wearability, and are more compatible with data acquisition circuits and algorithm processing on the backend [7,8,9]. Consequently, they have become an important approach in the current monitoring of physiological signals and actions, drawing extensive attention from researchers [10,11,12].

Fiber-based strain sensors, as important components of textile biomechanical sensors, exhibit advantages such as small size, light weight, fast response, and accurate detection of the applied strain on the attached object [13,14,15,16]. Researchers have designed various material structures to achieve strain-sensing performance [17,18,19,20]. The main strategy involves anchoring nanoscale safe and harmless conductive materials (such as carbon nanotubes (CNTs), Graphene, MXene) onto the surface or within the elastic material (fiber, porous material) through hybridization, impregnation, and assembly methods [21,22]. In fact, polyurethane (PU) has always been a popular matrix material for stretchable strain sensors as it is readily available and exhibits excellent stretchability. For instance, Li et al. conducted in situ polymerization of conductive polypyrrole on PU films, utilizing the mechanical mismatch between the conductive polymer and PU to achieve micro-crack formation through pre-stretching, thereby achieving outstanding tensile sensing effects [23]. Liu et al. fabricated PU nanofiber films via electrospinning and subsequently ultrasonically impregnated them with CNT to obtain a stretchable sensor [24]. Moreover, Baik et al. embedded silver nanoflowers into PU and polyester elastomer matrices to develop highly conductive stretchable strain sensors, which were successfully applied in human motion analysis [25].

The strain sensing is achieved by utilizing the reversible deformation and reconstruction of the conductive network formed by nanoscale conductive materials under cyclic strain, leading to changes in electrical resistance [26,27,28]. The sensing performance of the strain sensor depends on the transformation mechanism of the conductive network under strain [29,30]. It is evident that in order to obtain sufficient signal output, sensors need to generate stretching deformation over as much length as possible to ensure the change rate of the conductive network. Consequently, their application necessitates a greater degree of integrated space. Currently, most of the stretchable strain sensors adopt a sparse contact mode and increased resistance of the strain-induced conductive network. As a result, there is a competitive trend between the sensitivity factor and the sensitivity range of the strain sensor [31,32,33]. Moreover, the recovery of the conductive network after stretching heavily relies on the elastic force provided by the elastic material. Conductive networks with microcrack structures on the surface face the risk of incomplete resistance recovery [34,35,36,37].

The ancient structure of knots, which has been used by humans for thousands of years, can collect and concentrate the tensile forces acting on the ropes along the axial direction and transform them into pressure between the knots according to specific rules. The description of their topological mechanical properties has always been a hot topic in the field of micromechanics. In 2020, Dunkel used force-induced color-changing fiber materials to study the dispersion and energy states of internal forces in different knot structures. It was pointed out that as a strongly coupled system, the knot structure exhibits a ferromagnetic energy form similar to the Ising-type spin model with long-range interactions. The energy of topological fluctuations at the nodes is given by $\tau =\frac{1}{N}\sum_{i}{\left(q_{i}-\bar{q}\right)}^{2}=\tau_{0}\left(N\right)-\frac{2}{N^{2}}\sum_{i<j}q_{i}q_{j}$, where N is the crossing number, $\bar{q}=\sum_{i}q_{i}=W_{r}/N$ is the average twist, $\tau_{0}=1-1/N$ is the ground state energy density [38]. Therefore, by using knot structures to construct robust tensile strain sensors, it is possible not only to transmit tensile strain along the axial direction and convert it into pressure at the nodes, but also to realize the transition of the conductive pathway of the strain sensor from a sparse contact mode to a condensed contact mode. Furthermore, this type of tensile strain sensor based on knot structures no longer requires long-distance attachment and can even be achieved by conducting treatment at the knot structure. This is expected to innovate the structure and mechanism of tensile strain sensors [39,40].

In this research, a multi-layer polyurethane-fiber-prepared (MPF) sensor was fabricated by applying a CNT/PU sensing layer onto the surface of an elastic electrode using a wet-film method. The entangled topology of two MPFs was harnessed to convert stretching strain into pressure at the contact area, enabling the detection of stretching strain [40]. The high-modulus Ag/nylon threads orthogonally knitted onto low-modulus CNT/PU generate enhanced local stress concentration at the intersections and facilitate the amplification and transfer of the strain and pressure signals. The impact of coating mechanical properties and surface structure on strain-sensing performance was studied. A force regulator was introduced to control the mechanical properties of the entangled topology of the MPFs. By adjusting the thickness and length proportion of the force regulator, the sensitivity factor and range of sensitivity of the sensor could be managed, achieving a high sensitivity factor of up to 127.74 and a sensitivity range of up to 58%. Eight sensors were incorporated into a sensor array and integrated into a dance costume, effectively monitoring the multi-axis motion of the dancer’s lumbar spine. This presents a novel method for wearable biomechanical sensors.

## 2. Materials and Methods

Materials: Spandex (560 D, 840 D, 1160 D, 1680 D, purchased from Zhuji Haoting Chemical Fiber Trading Co., Ltd., Shaoxing, China), silver-plated nylon yarn (Ag/Nylon, purchased from Suzhou Tike Silver Fiber Technology Co., Ltd., Suzhou, China), carbon nanotubes (CNT, purchased from Shenzhen Turing New Materials Co., Ltd., Shenzhen, China), polyethylene glycol (PEG, molecular weight 2000, purchased from Shanghai National Medicines Group Chemical Reagents Co., Ltd., Shanghai, China), N,N dimethylformamide (DMF, purchased from Shanghai National Medicines Group Chemical Reagents Co., Ltd., Shanghai, China), polyurethane (PU, purchased from Guangzhou Boteng New Material Technology Co., Ltd., Guangzhou, China), and copper wire (0.2 mm, purchased from Dongguan Qianlong Nonferrous Metal Materials Co., Ltd., Dongguan, China).

Preparation of Elastic Core Shear Yarn: The spandex is uniformly pulled through a moving mechanical core-sheath equipment. The Ag/Nylon yarn is stabilized onto the outer surface of the spandex using a rotating winding method to produce the elastic core shear yarn.

Preparation of MPF: CNT, PEG, and PU are separately dissolved in DMF with mass ratios of 7:20:40:250. The mixture is mechanically stirred for 25 h and ultrasonically homogenized for 1 h to ensure uniform dispersion. The prepared solution is uniformly coated on the surface of the elastic core shear fiber using a coaxial coating method. The traveling speed of the elastic core shear fiber is 0.4 m/min, and the solution feeding rate is 0.4 mL/min. The coated multi-layered fibers are introduced into a pure water coagulation bath and then dried at 120 degrees to obtain the MPF.

Preparation of MPF Sensor: The MPF fiber is cut into segments. Cu wires are connected to the Ag/Nylon at the cut ends as electrodes and PU fibers are connected as force regulators. The two treated fiber segments are securely fixed in an entangled manner to create a sensor structure.

Method for Sensing Performance Testing: A Mark-10 (ESM-303) tensile-compression testing system is used in conjunction with a Keithley (2450) digital sourcing meter. The MPF is securely fixed to the gripping fixture of the tensile-compression testing system. The electrodes are connected to the digital sourcing meter using fine copper wires. Different levels of stretching amplitude and speed are set on the tensile-compression testing system, with a direct current voltage of 1 V applied to the sensor. The current variations of the sensor are monitored using the digital sourcing meter.

Method for Dance Movement Testing: Eight MPF sensors, each measuring 10 cm in length, are integrated into a dance costume. They are positioned roughly at the middle of the sternum, below the left front ribs, in the middle of the abdomen, below the right front ribs, at the middle of the thoracic spine (on the back), below the left rear ribs, at the middle of the vertebral column (lumbar vertebrae), below the right rear ribs, and at the upper and lower parts of the left and right ribcages. The extension cable of the Keithley 2450 is attached to both ends of each individual sensor’s MPF segment, with an input voltage of 1 V set. The dance performer wears the costume with integrated MPF sensors and demonstrates different dance movements on their body, performing five different dance movements for each sensor line. Finally, the Keithley 2450 is used to record the current variations of each MPF sensor corresponding to each dance movement, respectively.

## 3. Discussion

**Figure 1:**
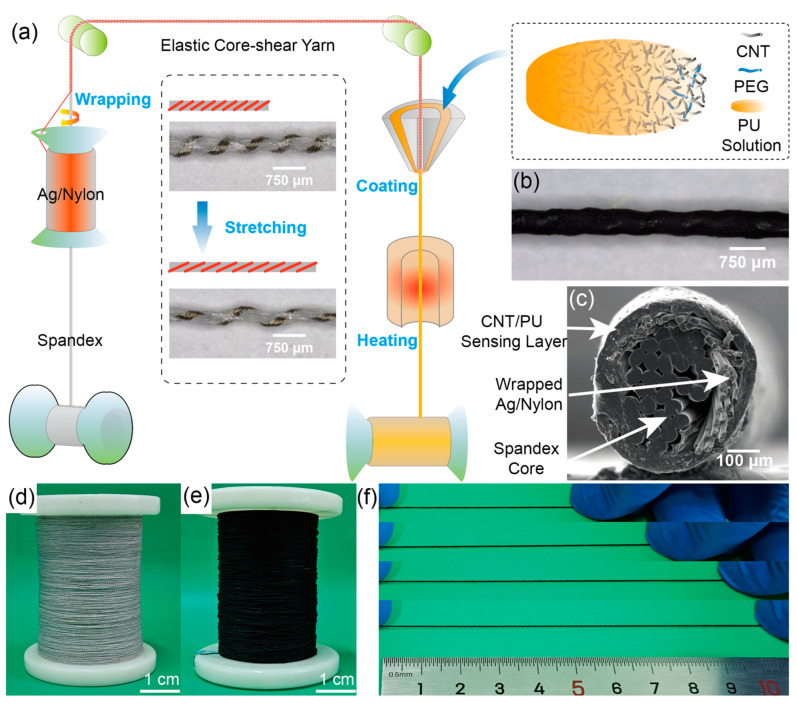
Preparation and morphology of MPF. (**a**) Schematic diagram of the preparation process of MPF, (**b**) Microscopic morphology of MPF, (**c**) Cross-sectional SEM image of MPF, (**d**) Elastic core shear yarn, (**e**) Digital photograph of MPF, (**f**) Photographs of MPF under different stretching states.

The schematic diagram presented in Figure 1 illustrates the process of preparing and characterizing the MPF. The MPF is composed of multiple layers of polyurethane and exhibits unique properties. To begin with, spandex is drawn into a mechanical core sheath device, while the Ag/Nylon yarn is spun and wrapped around the spandex to create the elastic core shear yarn. This wrapping imparts mechanical elasticity to the fabric, forming a spring-like structure on the spandex surface. The inset of Figure 1a shows the schematic diagram and optical microscope images of the elastic core shear yarn before and after stretching. It can be observed that the spandex diameter decreases when stretched, due to the Poisson effect, while the Ag/Nylon yarn remains tightly wrapped around it, resulting in an increased pitch of the spring-like structure. Next, the elastic core shear yarn is further processed in a coating apparatus, where a solution containing CNTs, PEG (polyethylene glycol), PU (polyurethane), and DMF (dimethylformamide) is evenly applied onto its surface. After solidification in a pure water bath and drying, the MPF is obtained. Figure 1b shows an optical microscope image of the MPF, displaying its uniform diameter of around 500 μm and a spiral morphology resembling the spring-like structure of the Ag/Nylon yarn. Figure 1c presents a cross-sectional SEM (scanning electron microscope) image of the MPF, revealing distinct layered structures. The core layer consists of spandex filaments serving as an elastic support layer, with a length of 300 μm. The intermediate layer is formed by the coiled Ag/Nylon yarn, which has a thickness of approximately 45 μm and is primarily responsible for signal collection. The outermost layer is the coated CNT/PU sensing layer, with a thickness of about 55 μm, showing even coating. Finally, Figure 1d and 1e provide images of the prepared elastic core shear yarn and the finished MPF, respectively. These images demonstrate the stable continuous preparation process and indicate the potential for batch production using this method.

**Figure 2:**
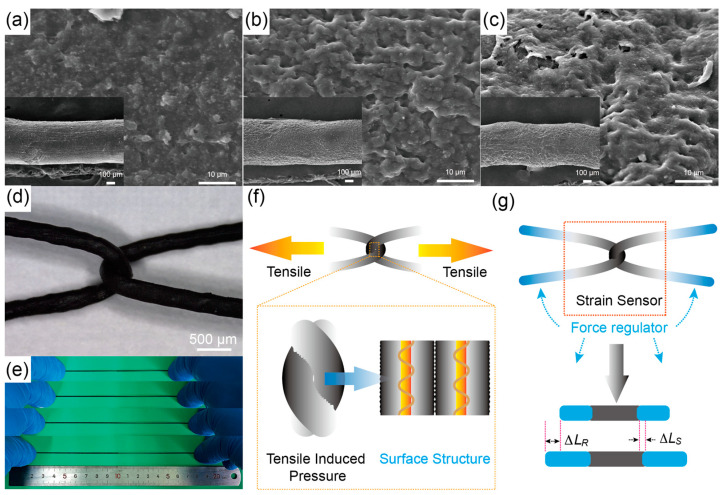
The surface microstructure of the MPF and the working mechanism of the entangled structural strain sensor. SEM image of MPF prepared with (**a**) 60 A PU, (**b**) 70 A PU, and (**c**) 80 A PU. (**d**) Optical microscopy image of the entangled structural strain sensor, (**e**) photos of the entangled structural strain sensor under different tensile states, (**f**) mechanism of electrical signal generation, and (**g**) working mechanism of the force regulator.

A different morphology on the surface of the CNT/PEG/PU/DMF solution coated onto the elastic core shear yarn was observed with PU prepared using 60 A, 70 A, and 80 A PU, as shown in Figure 2a–c. The surface of the MPF exhibited granular protruding structures, with larger particle sizes observed for MPFs prepared using PU with a higher modulus. This difference in particle size may be attributed to the varying concentration and viscosity characteristics of the dissolved PUs. The magnified SEM image in the top right corner of Figure 2b shows the surface of the MPF with added CNTs distributed randomly within the PU matrix, which is attributed to the lack of employing high-speed stretching or other processing methods that could induce CNT alignment. The resistances obtained from preparing MPF using 60 A, 70 A, and 80 A PU are 1.03 kΩ, 2.35 kΩ, and 2.94 kΩ, respectively. Figure 2d presents an optical microscope image of the entangled structure strain sensor, where two MPFs are in contact at the intersection. Due to the excellent elasticity of the MPF itself, the entangled structure strain sensor exhibits stretchability as well. Figure 2e illustrates photographs of the entangled structure strain sensor stretched at 20%, 40%, and 60% elongation. Based on the surface microstructure of the MPF revealed by the SEM images in Figure 2a–c, the mechanism of resistance variation in the entangled structure strain sensor can be speculated, as shown in Figure 2f. When assembled into a sensor, the complete conductive circuit is formed through the Ag/Nylon electrodes within two MPFs, the CNT/PU sensing layer, and the contact resistance between the CNT/PU layers. During the stretching process, corresponding pressure is generated at the intersection of two MPFs, leading to variations in contact resistance and tunneling resistance between the CNTs in the CNT/PU layers. As a result, the tensile-induced pressure can be obtained by measuring the resistance changes through the Ag/Nylon electrodes in the two MPFs. Additionally, a PU yarn with a different modulus was connected at the tail end of the MPF as a force regulator, as shown in Figure 2g. The different moduli between the MPF and the force regulator result in different deformations ($\Delta L_{S}$ and $\Delta L_{R}$) when the sensor is stretched. By adjusting the modulus and length of the force regulator, the ratio of $\Delta L_{S}$ to $\Delta L_{R}$ can be modified, enabling control over the output curve of the entangled structure strain sensor.

**Figure 3:**
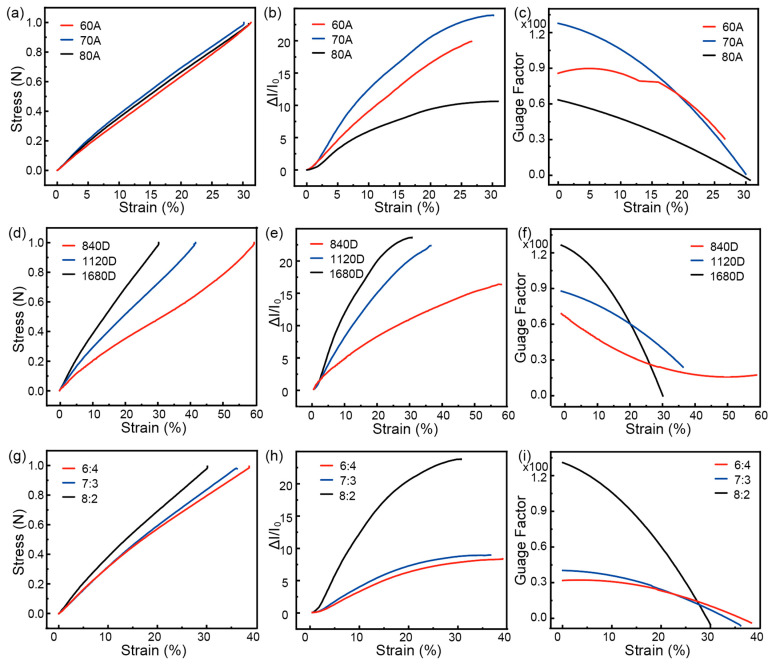
The mechanical and sensing performance of entangled structure strain sensors. The (**a**) stress–strain curve, (**b**) current variation curve, and (**c**) gauge factor for sensors prepared using different PU. The (**d**) stress–strain curve, (**e**) current variation curve, and (**f**) gauge factor for sensors utilizing different thicknesses of PU fibers as force regulators. The (**g**) stress–strain curve, (**h**) current variation curve, and (**i**) gauge factor for sensors with varying lengths of force regulators.

Figure 3 presents the mechanical and sensing performance of entangled strain sensors. In Figure 3a, the stress–strain curves of entangled sensors with CNT/PU sensing coatings prepared using different modulus PUs are shown. The results indicate that the mechanical performance of the sensors is not significantly influenced by the PU modulus. This is because the spandex in the MPF core layer bears the majority of the tensile stress. In Figure 3b, the electrical response of the sensors is shown. It is observed that the relative change in current of the sensors increases gradually with increasing strain. Comparing the responses of sensors prepared with different PU moduli under the same strain, it is found that the sensor prepared with 70 A PU has the highest relative change in current, followed by 60 A, and 80 A has the lowest. This is determined by the combined effect of the compressive deformation capacity of CNT/PU and the microstructure of the coating surface. Softer PU is more prone to compression deformation, resulting in larger changes in tunneling resistance and surface contact resistance. However, a rougher surface reduces the initial contact resistance while significantly increasing the surface contact resistance change during stretching. Overall, these findings provide insights into the relationship between the mechanical and sensing performance of entangled strain sensors. Figure 3c presents the gauge factor (GF) data of the sensors. GF is defined as $\frac{\partial (\frac{∆I}{I_{0}})}{\partial \varepsilon }$, where $\frac{∆I}{I_{0}}$ is the relative change in current of the sensor under strain ε. It can be observed that the GF values of the strain sensors show a decreasing trend with increasing strain. Among them, the sensor prepared with 70 A PU has the highest GF value (approximately 127.74), and its rate of decrease is also the fastest. The GF of the sensors reaches the lowest point at around 30% strain. The mechanical performance, strain-dependent relative current change, and gauge factor (GF) of the entangled sensor with different thicknesses of force regulator are shown in Figure 3d–f. The total length of the sensor is 10 cm, with 8 cm of MPF (made of 70 A PU) and 2 cm of FR. It can be observed from the graph that the deformability of the sensor significantly changes with the introduction of the force regulator. A lower diameter of the force regulator leads to higher strain required for the sensor to obtain the same stress. Consequently, the sensitive range of the entangled strain sensor is extended. However, the relative current change of the sensor decreases accordingly. By using an 840 D force regulator, the sensitive range of the sensor is extended to approximately 58%, with the maximum GF decreasing to only 70.04. With a 1120 D force regulator, the sensitive range is extended to about 36%, while the highest GF is 89.06. Figure 3g–i presents the mechanical performance, strain-dependent relative current change, and GF of the entangled sensor with different lengths of force regulator. The total length of the sensor is 10 cm, with a 70 A PU MPF and a 1680 D PU fiber force regulator. It can be observed that the effect of force regulator length on the regulation ability of the sensor’s GF and sensitive range is not as significant as modifying the force regulator diameter. Nonetheless, there is still a slight variation. A longer length of the force regulator leads to higher strain required for the sensor to obtain the same stress. When the ratio between MPF and force regulator length is 6:4, the sensitive range of the sensor only extends to approximately 35%. The performance comparison is provided in Table 1. It can be seen that among many different types of strain sensors, the MPF sensor can be continuously and stably prepared while exhibiting a considerable strain-sensing range and high GFs.

**Figure 4:**
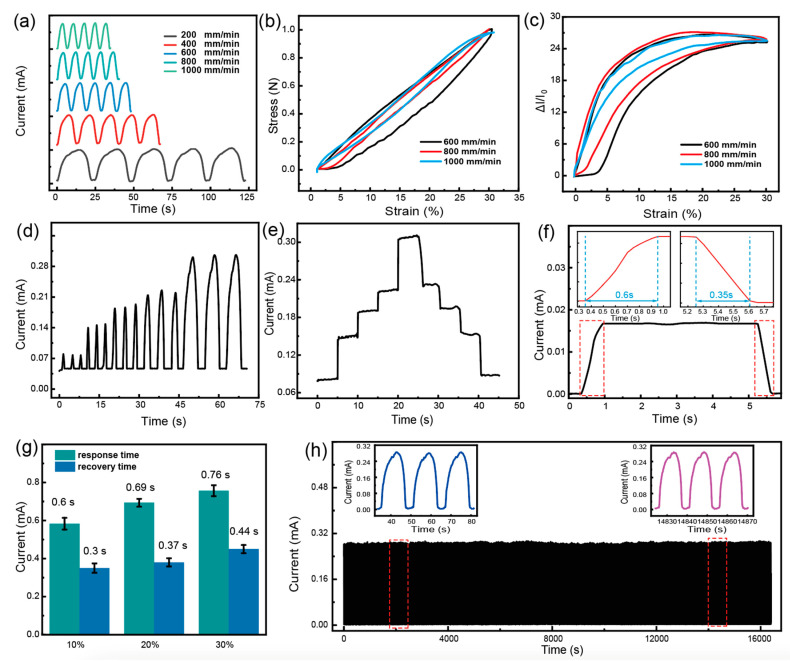
The sensing performance of entangled sensors. (**a**) The sensor outputs under different strain-applied speeds. The (**b**) stress and (**c**) relative current changes during the loading−unloading cycle with different strain-applying speeds. The current response of the sensor under (**d**) fast and (**e**) sustained strain. (**f**,**g**) The response time and recovery time of the strain sensors at strains of 10%, 20%, and 30%. (**h**) The cyclic stability of the sensors.

The recent experiments conducted on the strain sensor have provided further insights into its performance. These tests involved subjecting the sensor to cyclic stretching at a strain of 30% with varying strain-applying rates. The results, depicted in Figure 4a, demonstrate that the current response of the sensor remains consistent irrespective of the strain-applying rate. Moreover, the current response exhibits periodic variations that correspond to the applied strain rate, suggesting that the entangled sensor possesses remarkable dynamic stability. Figure 4b,c present the stress–strain hysteresis curve and electrical hysteresis curve under different strain rates, revealing that the mechanical hysteresis of the MPF sensor is relatively low due to the utilization of high-performance commercial spandex yarn as the raw material. However, its electrical hysteresis curve is relatively larger. With an increase in the applied strain rate, the hysteresis performance gradually improves. The maximum residual strain obtained from the tests is approximately 3.8%. In Figure 4d, the sensor was subjected to three repeated stretches at a strain rate of 800 mm/min and different strains. The findings indicate that, under the same strain, the sensor’s current response remains constant, showcasing its exceptional selectivity towards different strains. Additionally, Figure 4e showcases the sensor’s current response under various strain rates, confirming its ability to rapidly sense and maintain a stable current output under fixed strain conditions. Its current curve steadily returns during strain recovery demonstrating its stable electrical performance.

The response speed of the entangled strain sensor during rapid stretching and recovery processes under a strain of 10% is illustrated in Figure 4f. After the rapid application of strain, the output current of the sensor gradually transitions from a low platform to a high platform over approximately 0.6 s. Conversely, when the strain is removed, it takes about 0.35 s for the sensor’s output current to recover from the high platform to the low platform. The response speeds under 10%, 20%, and 30% strain were tested using the same method, and the results are shown in Figure 4g. The response times at 10%, 20%, and 30% strains were 0.6 s, 0.69 s, and 0.76 s, while the recovery times were 0.3 s, 0.37 s, and 0.44 s, respectively. It can be observed that as the strain increases, the response time exhibits a slight increase, yet still demonstrates the characteristic of faster recovery time compared to response time. Figure 4h presents the results of a durability test consisting of approximately 1400 cycles, showcasing the sensor’s variation in output current. The data demonstrates a constant change in output current, with minimal oscillations observed. This clearly indicates an excellent cycling stability of the strain sensor. Overall, these findings provide valuable insights into the performance and capabilities of the strain sensor, highlighting its potential for various applications.

**Figure 5:**
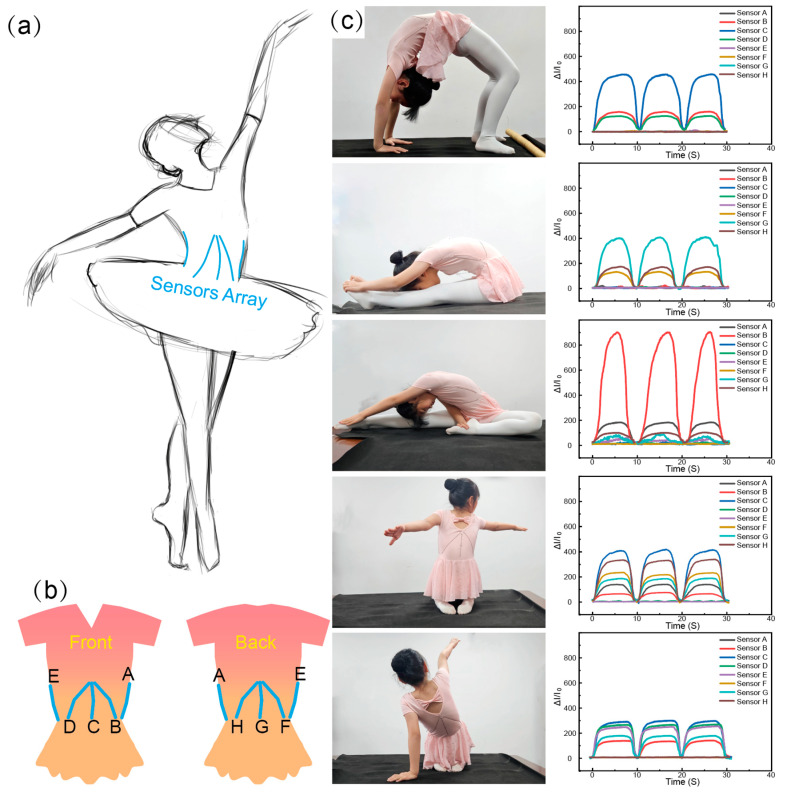
The application of the entangled sensor. (**a**) Illustration of utilizing sensor arrays to monitor the movements of the dancer. (**b**) The layout of strain sensors in the dance monitoring garment. (**c**) The various dance movements and respective current response of the sensor array.

The use of an entangled sensor in dance pose monitoring, as shown in Figure 5, demonstrates its application in capturing the movements of a dancer’s lumbar spine. The sensors are integrated into the lumbar spine region of the dance costume, as depicted in Figure 5a. Figure 5b illustrates the layout of the sensor array, consisting of eight entangled sensors. These sensors are strategically placed in the front and back of the waist region, with three sensors arranged in a dispersed manner from top to bottom to capture a wider range of movements. Additionally, one sensor is integrated on each side of the waistline, labelled as sensor A–H. In Figure 5c, a child dancer wearing the dance costume with the integrated sensor array is shown performing various dance movements. The diagram on the left depicts the sequence of movements, including lower waist movement, front leg extension, right leg extension, right leaning, and left leaning. On the right side of Figure 5c, the corresponding electrical signal responses of sensors A–H for each dance movement are presented. The child dancer repeated each dance movement three times, and the electrical signal response of the sensor array exhibited clear periodicity, with a high level of repeatability in each cycle. Furthermore, the study found that when the dancer performed movements involving only forward or backward motion along a single axis, only the three sensors integrated on the back or front side exhibited significant electrical signal responses. However, in more complex multi-axial movements such as leg extensions or side leaning with rotation, more than four sensors exhibited strong electrical signal responses. The successful application of the sensor arrays in capturing the lumbar spine movements of the dancer highlights its tremendous potential for various other applications. This technology could be used not only in dance pose monitoring but also in other fields where precise movement tracking is required, such as sports training, rehabilitation, and virtual reality gaming.

## 4. Conclusions

The MPF was created by applying a CNT/PU sensing layer to the outer surface of an elastic electrode using a wet-film method. The entangling structure of two MPFs was employed to convert stretching strain into localized pressure at the contact area, enabling the detection of stretching strain. The impact of coating mechanical properties and surface structure on strain-sensing performance was examined. A force regulator was introduced to manage the mechanical properties of the intertwined structure of the MPF. By adjusting the thickness and length ratio of the force regulator, the sensitivity factor and sensitivity range of the sensor could be adjusted, achieving a high sensitivity factor of up to 127.74 and a sensitivity range of up to 58%. Eight sensors were integrated into a sensor array and embedded into a dance costume, effectively monitoring the multi-axis motion of the dancer’s lumbar spine. This presents a fresh approach for wearable biomechanical sensors.

## Figures and Tables

**Table 1:** The performance comparison of the reported sensors.

Materials	Fabrication Method	Range	GF	Reference
Polyaniline/thermoplastic polyurethane-based blended composite fiber	Microfluidic spinning	0–50%	160	[41]
Multi-walled carbon nanotubes/graphene/silicone rubber/Fe_3_O_4_ nanocomposite	Silvered nylon	0–120%	8.43	[42]
		120–160%	100.56	
Elastic fabrics and carbon black/gelatin/polyurethane composite	Dip coating	0−5%	128	[43]
		5−30%	39	
Ecoflex/encapsulated carbonized silk	Carbonization process	0–200%	8.81	[44]
Polyvinyl alcohol/Poly3,4-ethylenedioxythiophene: polystyrene sulfonate hydrogel	3D printing	0–300%	4.07	[45]
Silvered nylon and PU/CNTs composite	Warp spinning, wet spinning	0–60%	127.74	This work

## Data Availability

Data are contained within the article.
